# Implementing the use of objective medication adherence data in routine clinical practice via the digital CFHealthHub platform: situation analysis and strategy development using the theoretical domains framework

**DOI:** 10.1186/s43058-022-00263-9

**Published:** 2022-02-08

**Authors:** Carla Girling, Anna Packham, Louisa Robinson, Madelynne A. Arden, Daniel Hind, Martin J. Wildman

**Affiliations:** 1 Clinical Trials Research Unit, University of Sheffield, Regent Court, 30 Regent Street, Sheffield, S1 4DA UK; 2 Department of Psychology, Sociology and Politics, Sheffield Hallam University, Collegiate Crescent, Sheffield, S10 2BQ UK; 3 Sheffield Adult Cystic Fibrosis Unit Sheffield Teaching Hospitals NHS Foundation Trust, Northern General Hospital, Herries Road, Sheffield, S5 7AU UK

**Keywords:** Cystic fibrosis, Adherence, Implementation, Theoretical domains framework, Intervention

## Abstract

**Background:**

Preventative inhaled treatments preserve lung function and reduce exacerbations in cystic fibrosis (CF). Self-reported adherence to these treatments is over-estimated. An online platform (CFHealthHub) has been developed with patients and clinicians to display real-time objective adherence data from dose-counting nebulisers, so that clinical teams can offer informed treatment support.

**Methods:**

In this paper, we identify pre-implementation barriers to healthcare practitioners performing two key behaviours: accessing objective adherence data through the website CFHealthHub and discussing medication adherence with patients. We aimed to understand barriers during the pre-implementation phase, so that appropriate strategy could be developed for the scale up of implementing objective adherence data in 19 CF centres.

Thirteen semi-structured interviews were conducted with healthcare practitioners working in three UK CF centres. Qualitative data were coded using the theoretical domains framework (TDF), which describes 14 validated domains to implementation behaviour change.

**Results:**

Analysis indicated that an implementation strategy should address all 14 domains of the TDF to successfully support implementation. Participants did not report routines or habits for using objective adherence data in clinical care. Examples of salient barriers included skills, beliefs in consequences, and social influence and professional roles. The results also affirmed a requirement to address organisational barriers. Relevant behaviour change techniques were selected to develop implementation strategy modules using the behaviour change wheel approach to intervention development.

**Conclusions:**

This paper demonstrates the value of applying the TDF at pre-implementation, to understand context and to support the development of a situationally relevant implementation strategy.

**Supplementary Information:**

The online version contains supplementary material available at 10.1186/s43058-022-00263-9.

Contribution to the literature

- Research indicates that the implementation of healthcare innovations may be more likely to succeed when context and theory are taken into consideration.
- In this study, healthcare professionals identified barriers to two behaviours that were key to the implementation of a national cystic fibrosis (CF) healthcare innovation. By coding barriers to the theoretical domains framework (TDF), a contextually relevant implementation strategy was developed, with a focus on clinician behaviour change.
- The study highlights the challenges CF teams face when implementing new remote monitoring of medication adherence and provides an important opportunity to apply the TDF in the pre-implementation phase of a healthcare innovation.

## Background

Cystic fibrosis (CF) is a long-term condition affecting 10,000 people in the UK. Although survival in the UK is rising, people with cystic fibrosis (PWCF) typically die from lung damage at a median age of 47 [1]. Preventative inhaled treatments preserve lung function by reducing infections [2–9]. Low adherence to these treatments is associated with exacerbations and decreases in lung function [10–15]. Despite the benefits of treatment, objectively measured adherence to preventative inhaled treatments is between 30 and 50% [16, 17]. Subjective self-report measurements—the norm in routine practice—substantially overestimate adherence rates [16]. As a result, low adherence is largely invisible to care teams, who are therefore unable to provide appropriate support to those who need it.

Without access to objective adherence data for inhaled medications it is difficult for clinicians to identify whether a patient is deteriorating due to non-adherence or due to novel pathology which requires a change of treatment. As disadvantaged populations have worse adherence and disease control than those from affluent areas, adherence is an equity issue and its support an ethical imperative [18]. To meet these challenges, we have worked with PWCF and healthcare practitioners to co-produce an online platform (CFHealthHub) [19, 20]. CFHealthHub displays real-time objective adherence data from dose counting nebulisers [21–24], allowing remote real-time monitoring of patient adherence. A national implementation exercise is now underway, supported by the NHS England commissioning for quality and innovation, in which objective adherence data will be embedded into routine CF care. In the first phase of this work, we have created a digital learning health system—a cohort study with research, implementation, and quality improvement functions—in three UK CF centres (ISRCTN14464661).

Many innovations successful in a single centre or trial fail to be adopted across healthcare organisations [25], particularly when the innovation is not adapted to the specific context [26] in different units. As such, implementation programmes need to identify the factors that influence the performance of the key behaviours that enable implementation, situated within the context of the target healthcare provider. An understanding of the interactions between context and behaviour, used in combination with theory, has the potential to optimise implementation strategy development [27–31].

Identifying the most appropriate theory for a given behaviour and context is challenging and choosing one theory over another may result in key determinants of behaviour being missed [32]. The theoretical domains framework (TDF) [33–35] is a synthesis of 33 different behaviour change theories, with 14 key domains that influence an individual’s Capability, Opportunity and Motivation to perform a behaviour (the COM-B model [36, 37]). To implement the use of objective adherence data in routine practice, clinical teams need to be able to perform two key behaviours (1) to routinely view patient’s objective adherence data and (2) discuss objective adherence with the patient. These behaviours might be expected to be sustained long term if they become established in local routines, with the hope that they become habitual [38]. By identifying barriers and facilitators to performing the behaviours, through the identification of TDF domains, linked to COM-B, potential reasons for implementation failure can be anticipated, understood, and addressed in advance.

The behaviour change wheel (BCW) is a tool to enable the design of interventions using a systematic approach that is underpinned by the COM-B model. Here, we report a detailed situation analysis [39], which used the TDF to identify potential pre-implementation barriers and facilitators of the desirable behaviours and inform the development of an implementation strategy using the BCW [37, 40].

## Methods

### Study design

This was a qualitative study using semi-structured interview data.

### Settings and participants

Participants (*n*=13) were healthcare practitioners from three participating CF centres, sampled from a combined multi-disciplinary team (MDT) of 125. A further seven healthcare practitioners were approached but were unable to participate due to time restrictions. At the time of this evaluation (April to August 2018), one centre had been involved in the development of CFHealthHub over 12 months and then started to use it in clinical practice and two of the three CF centres had taken part in a pilot trial of CFHealthHub as part of which one member of the MDT has been trained to use the CFHealthHub website and to deliver the intervention. All sites were therefore within the early stages of implementation. The CF population for the centres covered large geographical areas, across multiple counties. Each centre supported between 175 and 250 PWCF, at the time of the study.

We purposively sampled from the MDT based on centre and professional category (Table 1). Recruitment continued until the researchers determined that data saturation had been met, as defined by ‘informational redundancy’ [41], whereby no new comments were identified in the interviews.

**Table 1:** Participants by profession and centre

Profession	Centre 1	Centre 2	Centre 3	Total
Consultant Respiratory Physician	2	2	1	6
Physiotherapist	1	1	0	2
Nurse	1	1	1	3
Local CFHealthHub Lead	0	1	1	2
Counsellor	1	0	0	1

### Procedure

The study team contacted healthcare practitioners by email. All participants gave informed consent prior to the interview. Interviewers (CG, AP and DH) were known to three participants through wider project work but were not from the same institution. The interview topic guide (Additional file 1) was based upon TDF constructs [40] focusing on the behaviour of accessing adherence data through CFHealthHub (1) and discussing adherence with patients as part of routine practice (2). Not all questions were relevant to all participants; for example, at the time of the interviews, the use of data to benchmark quality of care between centres was not yet available to all members of the clinical team. The interview guide was developed by investigators with expertise in behaviour change (MA) and cystic fibrosis (MW) and piloted by one clinical member of staff. Interviews were conducted face to face or via a telephone. The duration of interviews was between 17 and 55 (mean 37) min. All interviews were digitally recorded and transcribed verbatim and imported into Nvivo 12 (QSR International).

### Analysis

Transcripts were analysed using framework analysis [42] based on the TDF. Two researchers double-coded each interview, where fragments were coded to more than one domain these were cross-indexed. Researchers met regularly to discuss coding, data saturation, and reach consensus on discrepancies.

### Implementation strategy development

Based on the findings of the framework analysis, members of the team, including a respiratory physician (MW) and health psychologists (MA) developed an implementation strategy using the behaviour change wheel (BCW) approach [37]. Firstly, a behavioural needs analysis was completed for the behaviours of routinely accessing objective adherence data (1) and discussing adherence (2) (Table 2). Interview data, mapped to TDF domains, were used to perform a behavioural diagnosis for behaviour #1 and #2, allowing the researchers to identify what needs to change in order for the behaviours to be routinely performed. From here, intervention functions were identified. Intervention functions are broad categories, linked to the COM-B model. Within each intervention function there are multiple possible behaviour change techniques (BCTs). To ensure context-based decisions on intervention content and delivery, the APEASE guidelines were applied to each of the nine potential intervention functions, specified in the BWC. This allowed the researchers to evaluate functions for affordability, practicality, effectiveness, acceptability, side effects/safety, and equity. Having identified the barriers and relevant intervention functions, behaviour change techniques (BCTs) were selected. These are displayed as implementation ‘strategy’ modules.

**Table 2:** COM-B model components and preliminary behavioural needs analysis

COM-B model component	Behaviour 1: *Opening CFHealthHub and accessing adherence data through the ‘How am I doing?’ page for each patient.* What needs to happen?	Behaviour 2: *Discuss adherence with patients using the non-judgemental CFHealthHub style.* What needs to happen?
**Physical capability**	- Have the skills to be able to use CFHealthHub correctly, for example interpreting objective adherence graphs	- Have the skills to be able to use CFHealthHub correctly, for example interpreting adherence graphs and speak to patients appropriately about adherence
**Psychological capability**	- Understand the importance of adherence to nebulisers in CF - To understand what patients’ adherence is across their centre - Be able to remember to use CFHealthHub to access adherence data - Be able to self-monitor use of CFHealthHub	- Understand the importance of adherence to nebulisers in CF - Be able to remember to use non-judgemental adherence language
**Physical opportunity**	- Have the resources to access objective adherence data e.g. a computer, internet, CFHealthHub log in - Have time to access objective adherence data on CFHealthHub	- Have the resources to access objective adherence data e.g. a computer, internet, CFHealthHub log in - Have a realistic plan of when to deliver adherence discussions - Have the time in the working day to deliver adherence discussions
**Social opportunity**	- Be/feel supported by the CF team to use CFHealthHub - Have senior colleagues/management endorse and use CFHealthHub	- Be/feel supported by the CF team to use CFHealthHub
**Reflective motivation**	- Perceive adherence data as a part of their clinical role - Perceive few/no concerns about using CFHealthHub with patients - Believe that objective adherence data can improve patient care - Intend to use CFHealthHub and objective adherence data	- Perceive adherence support as a part of their clinical role - Perceive few/no concerns about using CFHealthHub with patients - Believe that adherence discussions can improve patient care - Feel confident in using CFHealthHub and discussing adherence - Want to achieve better patient adherence as a centre
**Automatic motivation**	- Have an established routine within the clinical workplace for accessing objective adherence data - Have a habit of opening objective adherence data in every clinical encounter	

### Ethics

After review from the study’s Patient and Public Involvement group, ethical approval was obtained from London-Brent Research Ethics Committee (ref 17/LO/0032).

## Results

We present a summary of data by theoretical domains, ordered by the umbrella concepts, capability opportunity, and motivation (Table 3) before discussing the development of the implementation strategy. We have combined the data for the two behaviours of focus.

**Table 3:** Theoretical domains framework (TDF) illustrative quotes

TDF domain	Example quote	Interview ID
**Knowledge**	I know that self-reported data is a lot … higher than actual data and as clinicians we get it wrong as well, we like, overestimate it. Erm, and that just that it’s so important for … maintaining their health so we know that like they’re likely to stay better if their adherence is better to their nebulisers.	S01F03
**Skills**	…I’m not sure I do really, (yeah), so I kind of think I do (yeah) and I try very hard to not be judgemental and I try very hard not to give peoples plans, but to help people make their own plans and to discuss ideas and habits with people rather than telling people what to do. But, I did think yesterday, when I was having this conversation maybe I ought to be doing this training because I’m not sure I’m as skilled as I ought to be.	S02F03
**Memory, attention and decision-making processes**	So on the ward is much more ad-hoc and it might come up in a ward round, and it might come just... I float round and stick my head round the door and talk to patients without the rest of the team sometimes... it could be completely ad-hoc or I could go with an agenda that is specifically to talk about adherence ‘cause they’re rubbish.	S01F02
**Behavioural regulation**	No, I don’t think we have a set way to remember to use it, I mean I know certainly that physios should be looking at that, and I think the doctors are but in terms of other clinicians, I’m not sure they necessarily are.	S02F05
**Social/professional role and identity**	I think particularly the physios, the doctors. I mean it’s helpful for everybody but, I think, in practice, we should all… all clinicians should look at it but I think in practice the ones who tend to look at it are the doctors the physios and the nurses.	S02F05
**Beliefs about capabilities**	I: How confident do you feel discussing adherence with patients? P: Mm, well, people can be confident and be really bad at it (I laughs), but I’m increasingly confident about talking about it and increasingly surprised about the some of the discussions that I get into with even people who I thought they’re quite good at doing their treatment and sometimes you don’t get it on the first second, third, fourth attempt it suddenly spills out sometimes by mistake or sometimes they just fess up so I’m getting better at it for sure but whether I’m any good I couldn’t tell you.	S01F02
**Optimism**	I’d like to say I’m confident but I’m, I don’t know cause of you know people are people and well we all know that, you know, everyone has their lives… I don’t necessarily think, but I’d like to think that it would make some difference at least	S02F04
**Beliefs about consequences**	I think actually if the team don’t necessarily use it in the right way it could actually just be used as something to just tell patients off with.	S03F01
	but if you can get people to sort of believe that actually it’s worth it because in the long run it actually will decrease your workload and actually make the patients better and probably using your service less…	S02F02
**Intentions**	I: Have, have you made a decision to use CFHealthHub and to discuss adherence? P: No, not particularly, as I said we didn’t have a lot of, I know something going on but as I said I won’t tell you, I know the importance of it but no probably not a lot done from my perspective.	S03F02
**Goals**	I think the aspiration has got to be that that is just normal. That’s just you know lung function, weight adherence data and it just something that we have that we look at automatically and it something that the patient’s own as well. That they have that information, so they have all those metrics together and there’s some way that we can react to that outside of a clinic setting…	S03F03
**Reinforcement**	I think, you know, if someone’s really struggled and then they’re suddenly on board, the I think the that’s that just makes you feel really pleased for them.	S01F04
**Emotion**	I think, yeh, it does spark a bit of an emotion, and I don’t even know all the patients that well as I’m quite new to the service. So but I think it is, you know, you feel a little bit, a bit shocked I suppose. Although it shouldn’t be shock, because I know patients do struggle, but you know, it is quite shocking. And I think the team will be really shocked when they do have access to CF Health Hub to actually see the scale of the problem.	S03F01
**Environmental context and resources**	Probably mainly staffing, and I say that because we have this electronic patient record so we needed to get our computer infrastructure sorted so we actually have enough computers to... to use HealthHub.	S01F02
**Social influences**	…we invited erm the lead for respiratory and a nurse matron and various other people to come and meet and speak about it and they didn’t take that up, we do discuss it regularly at our management meeting where we do have respiratory business management representation. But I think there is scope …you know for us to flag it at a higher level within the trust to sort of say you know, to shout about it really	S03F03

### Domains related to capability

Overall, participants had some knowledge of the concept of nebuliser adherence, both in relation to the challenges of nebuliser adherence and the impact on lung health. The disparity between subjectively reported and objectively measured adherence was also frequently reported. By comparison, knowledge about CFHealthHub was variable between centres and healthcare practitioners; some consultants were unaware of patient facing CFHealthHub content but were aware that the platform displays adherence data. The most engaged participants demonstrated a more insightful understanding, describing patient-specific content and its use for both patients and healthcare practitioners:

“…so it’s a monitoring tool I’d say as well as supportive tool for adherence” (S01F04).

Most participants reported no formal training in using CFHealthHub or in how to discuss adherence with PWCF. Participants used skills from their professional training when discussing adherence with patients:

“…I do it, I suppose in my own counselling type way…” (S01F01).

Furthermore, skills and training influenced participants’ willingness to discuss adherence. Participants with backgrounds in counselling and training in motivational interviewing reported this as a facilitator for discussing adherence. Participants reported remembering (memory), paying attention, and decision processes in using adherence data as effortful. Discussing adherence with a PWCF was only done when prompted by conversations with PWCF in clinic appointments, for example during changes in prescribed medication. Even when adherence was remembered, participants did not necessarily access the objective data from CFHealthHub. No one reported formal behavioural regulation strategies to ensure adherence was discussed with PWCF. Where CFHealthHub was accessed as a team, this was during MDT meetings and was driven by specific individuals (see opportunity).

### Domains related to opportunity

TDF domains relating to both physical and social opportunity featured prominently in the sample. Participants described the physical barriers relevant to their centre’s environment, such as the availability of clinic rooms in which to deliver adherence support. Access to computers and the Internet also impeded the ability to open the objective adherence data on CFHealthHub. All participants described time as being a significant barrier for talking to PWCF about medication adherence (#2) and opening the objective adherence data at meetings or with colleagues (#1):

“…meetings are quite quick and then there are other things that we need to talk about and we don’t necessarily have time to factor in the adherence in it in a detailed way”. (S01F02)

Participants thought that physiotherapists and nurses had more contact time with patients than consultants and therefore had more time to use CFHealthHub (links to professional role).

In addition, all centres reported that limited staff capacity, particularly during the winter months, was a barrier to both behaviours. However, participants did note that CFHealthHub would make accessing adherence data easier than previous systems^1^. At least one influential figure or ‘CFHealthHub Champion’ from each centre was identified. A factor that appeared to be associated with social influences was how passionate the individual was about CFHealthHub. Participants also felt that doctors and consultants had the most influence in centres and were an important factor in the adoption of adherence data into practice. Some participants reported feeling that they were not individually able to implement change in their centre; they believed change would require team effort.

### Domains related to motivation

Professional role featured heavily as a theme in determining who accessed CFHealthHub and which individuals in a centre provided adherence support to patients. Those who reported they did access the objective adherence data on CFHealthHub and have adherence discussions did so because ‘it is part of my job isn’t it?’ (S03F01). Each team reported an individual as being responsible for opening adherence data. This meant that the behaviour of opening adherence data fell down when the individual was unavailable 'We planned having it in every MDT meeting, … we feel slightly guilty when [name] isn’t here ‘cause she’s the one who usually sets that for us’ (S01F02).

Participants reported the goal that CFHealthHub would be used routinely in MDT meetings, but had varying levels of intentions to use CFHealthHub. Physiotherapists reported that they intended to access adherence, whereas Consultants generally stated they might access adherence data themselves or through a colleague. However, Consultants report that they did not have intentions of performing the second behaviour of delivering adherence support.

“I don’t think I use it with patients, cause I don’t think I have the time to sit and use it with patients; plus it’s mainly delivered probably by the physios (yeah) at the moment in our service, or [name] might be working with one or two. So, because that you already know that they’re doing with it, I’ll talk about their adherence, but I’m not gonna, I don’t sit down with (and open it up)” (S01F05).

Participants discussed a range of beliefs about the power of adherence data and CFHealthHub and the consequences of using these tools. Although these was a consensus that using CFHealthHub and discussing adherence could improve patient care and could increase adherence, participants felt this would be limited to certain groups of patients or that they would see small incremental changes ‘…If you get people who are at 30% up to 40% that’s good and if you get people who are at 55% up to 65% that’s good and that’s where I think the benefit’s going to be’ (S02F03). In addition, healthcare practitioners believed that adherence data would not be helpful for patients with complicated home lives. The circumstances and their willingness to engage with adherence were also reported as barriers to supporting adherence, suggesting that they were reluctant to discuss this with everyone.

“I suppose both depending on what sort of place they’re in at that time, how their mood is… Some people we’re building relationships with when they first come to us and we don’t want to be heavy....” (S01F01)

Some participants perceived negative consequences. These participants were concerned objective adherence data could become a tool for “telling off” patients. This belief was influenced by the knowledge that that the clinical team had not received adequate training in accessing and using adherence data. This was thought to impact on the capability of the team to use the data in a positive, patient-centred way.

“… I think without the right training and support with the team is that actually it could then be used in a negative way with the patient. So actually as a tool to tell patients off …” (S03F01).

Participants received some reinforcement for using CFHealthHub. Viewing improvement in patient’s adherence was felt to be rewarding. Participants also reported positive emotions when seeing improvements in PWCF adherence.

### Implementation strategy development

All 14 domains were relevant to routinely access objective adherence data (#1); six were relevant to discussing adherence with patients (#2) (Tables 4 and 5). Intervention functions defined in the BCW were considered in relation to the behavioural needs assessment (Table 2) and reported barriers (Tables 4 and 5). Discussions around the implementation intervention considered the needs of different professional roles, with the reported barriers to performing each of the behaviours. The intervention functions were evaluated using the APEASE guidelines, which led to the rejection of three intervention functions, as likely to be impractical (restriction), ineffective (incentivisation) or unacceptable (coercion), see Table 6 for specific reasoning. Table 7 provides more detail on the selected proposed intervention functions that would go on to help identify suitable BCTs. Six intervention functions were selected by the researchers as suitable (training, education, environmental restructuring, enablement, modelling and persuasion), based on the needs assessment (Table 2). The intervention functions were further defined, leading to the selection of 31 specific functions for routinely accessing objective adherence data (#1) and 12 for discussing adherence (#2) (Table 7). Each BCT was discussed in relation to the two behaviours and identified barriers. This led to the identification of BCTs that the researchers deemed useful. The practicality, resources and expertise from the central study team were also considered when selecting and grouping the BCTs into potential implementation modules (Tables 8 and 9).

**Table 4:** Behaviour 1—TDF to expand COM-B components using interview data

COM-B component	TDF domain	Relevance of domain	Behaviour 1 Intervention?
Physical capability	Physical skills	• Receive direct training of CFHealthHub • Receive ongoing support for CFHealthHub skills • Being able to interpret objective adherence data • Being able to navigate CFHealthHub	Yes: develop skills for using CFHealthHub and have these regularly refreshed
Psychological capability	Knowledge	• Understand patients’ overall adherence in the centre • Identify which patients struggle with adherence • Understand what features are on CFHealthHub • Understand how to add information to CFHealthHub e.g. prescription updates	Yes: develop knowledge about centre adherence and individual patient adherence. Develop knowledge of CFHealthHub and theory behind it
	Memory attention and decision processes	• ‘Forgetting’ when busy • Only remembering when prompted by discussion with patient or clinician	Yes: notice and remember to open CFHealthHub with every patient.
	Behavioural regulation	• MDT meeting as a cue to action • Data improvements as feedback • No routine	Yes: develop skills of goal setting, action planning and enable self-monitoring
Physical opportunity	Environmental context and resources	• Having a laptop or computer • Having Wi-Fi available • Regular meetings to open and share adherence data	Yes: Alter structure of centre to accommodate CFHealthHub. Problem solve time and space issues.
Social opportunity	Social influences	• Lack of support from senior management • Lack of support from other team members • Not having a CFHealthHub champion	Yes: Facilitate support from others via problem-solving
Reflective motivation	Professional/social role and identity	• Deferred responsibility for understanding patient adherence “it isn’t part of my role”	Yes: links to knowledge and skills, educate/demonstrate usefulness of CFHealthHub for all roles. Perceive adherence support as a crucial part of all CF care.
	Beliefs about capabilities	• Not confident in how to interpret objective adherence charts	Yes: increase perceptions of capability.
	Optimism	• Opening objective adherence data at every encounter is not achievable	No
	Beliefs about consequences	• Objective adherence data will be used to tell patients off • Belief that embedding will require a lot of staff energy	Yes: develop appropriate beliefs about necessity of adherence data, address concerns.
	Intentions	• Low intentions within teams, specifically no intention of doing the behaviour if not related to role (see professional role)	Yes: links to professional role and identity, links to skills and knowledge.
	Goals	• To improve patient care • To improve patient health	No.
Automatic motivation	Reinforcement	• There’s no immediate reward for using objective adherence data • CFHealthHub can show threatening information (links to emotions)	Yes: provide reward for change through individual and centre level feedback. Use threatening information as a mode for change.
	Emotion	• Stress caused by overall workload • Cognitive dissonance caused by threatening data (see reinforcement)	No. Address environmental issues to reduce stress.

**Table 5:** Behaviour 2—TDF to expand COM-B components using interview data

COM-B component	TDF domain	Relevance of domain	Behaviour 2 Intervention?
Physical capability	Physical skills	• Receive direct training of CFHealthHub • Receive ongoing support for CFHealthHub skills • Being able to interpret the adherence data • Being able to navigate CFHealthHub • Having the skills to discuss adherence	Yes: develop skills for discussing adherence in a non judgemental manner and have these regularly refreshed
Psychological capability	Knowledge	• Understand patients’ overall adherence in the centre • Identify which patients struggle with adherence • Understand what features are on CFHealthHub • Understand how to add information to CFHealthHub e.g. prescription updates	Yes: develop knowledge about centre adherence and individual patient adherence. Develop knowledge of CFHealthHub and theory behind it.
	Memory attention and decision processes	• ‘Forgetting’ when busy • Only remembering when prompted by discussion with patient or clinician	Yes: have a system in place to notice adherence and arrange patient support
	Behavioural regulation	• Data improvements as feedback • No routine	No
Physical opportunity	Environmental context and resources	• Having a laptop or computer • Having Wi-Fi available • Having a clinic room available • Time to delivery adherence support	Yes: Alter structure of centre to accommodate adherence support. Problem solve time, space and technology issues.
Social opportunity	Social influences	• Lack of support from senior management • Lack of support from other team members	No
Reflective motivation	Professional/social role and identity	• Supporting adherence seen as a physio role	No
	Beliefs about capabilities	• Low confidence in adherence support increasing adherence for all patients • Patients’ willingness to engage with the team • Some staff would be confident but bad at discussing adherence	Yes: increase perceptions of capability.
	Optimism	• CFHealthHub improving patient health is achievable • Improving patient health is not achievable for all patients	No
	Beliefs about consequences	• Patients with complicated home lives won’t be effected • Objective adherence data will be used to tell patients off • There will only be small incremental changes for participants • Belief that embedding will require a lot of staff energy	Yes: develop appropriate beliefs about necessity of adherence support, address concerns
	Intentions	• N/A	No
	Goals	• To improve patient care • To improve patient health	No
Automatic motivation	Reinforcement	• There’s no immediate reward for using CFHealthHub • Improved lung function of patients visible • CFHealthHub can show threatening information (links to emotions)	Yes: reward change through feedback
	Emotion	• Stress caused by overall workload • Cognitive dissonance caused by threatening data (see reinforcement)	No

**Table 6:** Potential intervention ideas rejected through APEASE

Potential intervention focus	Potential intervention function	Reason for rejection (APEASE)
Create an expectation of increased cost to centre for not engaging	Coercion	Not acceptable to staff
Limit time spent on patient rescue	Restriction	Not practical as there are no options to restrict and potential side effects
Provide tangible financial gains for the centre	Incentivisation	Not likely to be effective in the staff who should be performing the behaviour, although may have an overall impact on the centres participation in the implementation project.

**Table 7:** Proposed intervention functions

COM-B	TDF domain	Proposed intervention functions	Specific functions: behaviour 1	Specific function: behaviour 2
Physical capability	Physical skills	Training	• Skills training in CFHealthHub use	• Skills’ training in adherence support
Psychological capability	Knowledge	Education	• Educate about CFHealthHub and habit formation • Educate about patient adherence	• Educate about non-judgemental adherence support
	Memory attention and decision processes	Environmental restructuring, education, training, enablement	• Train to remember to use CFHealthHub • Restructure environment to provide memory cues	• Enable referral system for patients requiring adherence support
	Behavioural regulation	Enablement training	• Train to set goals, action plan, self-monitor, make habits • Identify prompts and cues in the environment (creating habits) • Providing self-monitoring adherence data through click analytics • Agree a goal e.g. using CFHealthHub in X number of consultations and discuss and problem solve the barriers faced.	N/A
Physical opportunity	Environment	Environmental restructuring, enablement	• Add in cues to prompt behaviour • Problem solving of environmental barriers • Plan performance of the behaviour in the clinical setting.	• Problem solving of environmental barriers
Social opportunity	Social influence	Environmental restructuring, modelling, enablement	• Influential centre figures to demonstrate use of CFHealthHub in training and during regular team meetings. • Practical social support e.g. support network of trained colleagues to shadow. • Regular teleconferences between peer groups to discuss adherence data • Reflect on past successes as a team during regular team meetings.	• Practical social support e.g. support network of trained colleagues to shadow. • Reflect on past successes as a team during regular team meetings.
Reflective motivation	Social/professional role	Modelling, education, persuasion	• Each professional role to have a ‘change agent’ demonstrating the behaviour. • Credible sources of information about supporting adherence and the benefits sought for different professional groups. • Information from others about the usefulness of objective adherence data	• Information from others about the usefulness of CFHealthHub
	Beliefs about capabilities	Modelling, education, persuasion, enablement	• Provide information about moving from rescue to prevention, persuade others that this is achievable.	• Provide information about moving from rescue to prevention, persuade others that this is achievable.
	Beliefs about consequences	Modelling, education, persuasion, enablement	• Illustrate that patients will not run away from the clinical team • Publications, RCT results, information from pharmacy (e.g. about changing prescription regimens) in favour of adherence data.	• Demonstrate that supporting adherence can increase adherence with case studies • Lead interventionist to monitor adherence discussions (shadowing or record sessions) to review quality and redo training where necessary.
	Goals	Modelling, education, persuasion, enablement,	• Support to set individual behaviour goals. • Support whole centre to decide on goals. • Address conflicting goals	• Support to set goal to deliver adherence support to patients who request it
	Intentions	Modelling, education, persuasion, enablement	• Information (relevant to role) about health consequences for patients, information about time/money savings if used properly. See social/professional role.	• Encourage intentions to deliver adherence support
Automatic motivation	Reinforcement	Training,	• Patient stories of positive case studies (natural reward)	• Patient stories of positive case studies (natural reward)
	Emotions	Modelling, enablement, persuasion	• Feedback on behaviour • Feedback on outcome of behaviour • Reduce threat of viewing low adherence on CFHealthHub	• Reduce threat of viewing low adherence on CFHealthHub

**Table 8:** Intervention modules proposed for behaviour 1

Module	COM-B	Intervention functions	Proposed BCTs
**Training package: CFHealthHub training and adherence support training:** Training resources, refresher training, shadowing, fidelity assessment by lead interventionist	Physical capability Psychological capability Social opportunity	Training Education Persuasion	• 5.3 Information about social/enviro consequences • 6.1 Demonstration of the behaviour • 8.1 Behavioural practice and rehearsal • 9.1 Credible sources • 3.2 Social support (practical) • 6.3 Information about others approval
**Quality improvement cycles:** plan do study act cycles and continuous metrics feedback	Physical opportunity Reflective motivation Automatic motivation	Education Environmental restructuring Enablement	• 12.1 Restructuring physical environment • 1.2 Problem solving • 2.2 Feedback on behaviour • 6.2 Social comparison • 4.4 Behavioural experiments
**Improvement collaborative group:** Peer support, multicentre events, share success stories, problem solve together	Reflective Motivation Social Opportunity	Persuasion Modelling Enablement	• 6.1 Demonstration of behaviour from influential figures • 6.3 Information about other approval • 3.1 Social support (unspecified) • 6.2 Social comparison • 2.2 Feedback on the behaviour • 1.2 Problem solving • 15.3 Focus on past success
**Planning, routine and habits:** Goal setting, identifying prompts/cues, action planning	Psychological capability Reflective motivation	Training Enablement Environmental restructuring	• 1.1 Goal setting • 7.1 Adding prompts/cues • 1.4 Action planning • 1.5 Review behaviour goals • 8.3 Habit formation

**Table 9:** Intervention modules proposed for behaviour 2

Module	COM-B	Intervention functions	Proposed BCTs
**Training package: CFHealthHub training and adherence support training:** Training resources, refresher training, shadowing, monitoring by others with feedback	Physical capability Psychological capability Social opportunity	Training Education Persuasion	• 6.1 Demonstration of the behaviour • 8.1 Behavioural practice and rehearsal • 9.1 Credible sources • 3.2 Social support (practical) • 6.3 Information about others approval
**Quality improvement work:** plan do study act cycles and continuous metrics feedback	Physical opportunity Reflective motivation Automatic motivation	Education Environmental restructuring Enablement	• 12.1 Restructuring physical environment • 1.2 Problem solving • 2.2 Feedback on behaviour
**Improvement collaborative group:** Peer support, multicentre events, share success stories, problem solve together	Reflective Motivation Social Opportunity	Persuasion Modelling Enablement	• 6.1 Demonstration of behaviour from influential figures • 6.3 Information about other approval • 3.1 Social support (unspecified) • 1.2 Problem solving • 15.3 Focus on past success

### Implementation strategy modules

Tables 8 (#1) and 9 (#2) demonstrate how intervention functions, identified from barriers reported in interview data, link to proposed BCTs. On this basis, we have put together the following modules:

### Educational/training package

(#1) To address the varied training and education barriers to routinely using objective adherence data, a face to face and online educational training package would be provided, with the aim to provide practical support, such as instructions on how to perform the behaviours and information about others approval of the behaviour, for example local endorsement by members of the clinical team. As participants reported a varied understanding of the relevance of objective adherence data to CF care, the training package would focus on presenting information about the consequences of non-adherence for the PWCF and the relevant impact on the clinical team. To address social influence barriers such as lack of senior management support, it is important that a Consultant Physician provides demonstrations of the behaviour during these sessions and endorses accessing adherence data (#1), alongside credible sources of information and the opportunity to rehearse the behaviours.

(#2) The same module principals should be applied, however focused on delivering non-judgemental conversations and behaviour change techniques, as described in CFHealthHub intervention development [43, 44].

### Quality improvement cycles

(#1) Quality improvement (QI) methods were identified as a potential strategy to address centre specific barriers and provide feedback on the frequency of the performance of the behaviours at an individual and centre level. QI can be used to perform a number of BCTs (see Table 8) and is adaptable to individual centre context [45]. The implementation will use The Dartmouth Institute QI methodology, allowing the BCT problem solving, through process mapping and Plan Do Study Act (PDSA) cycles. These techniques will allow small behavioural experiments for change to be implemented, measured (providing feedback) and revised as required. As well as supporting centres to address environmental issues and improving the efficiency of the team, this strategy could be used to address specific concerns. For example, where the belief in prescription accuracy is a barrier, then the team would use quality improvement PDSA cycles to address their centre prescription processes and integrate updating CFHealthHub prescriptions that drive adherence data accuracy, into current practices. QI should be data driven [46, 47] to provide measurable feedback. To overcome the lack of behavioural regulation, healthcare practitioners should be given feedback on their own performance of behaviour #1 through frequency of website clicks into objective adherence data, collected as part of PDSA cycles.

(#2) Relevant CFHealthHub page clicks should be fed back to healthcare practitioners delivering adherence discussions. Barriers identified such as clinic space and time can be addressed through QI cycles, in the way described for #1.

### Cystic Fibrosis Improvement Collaborative as an improvement support module

Creating an improvement collaborative would link CF centres into a community of practice and provide a platform for sharing past successes and learning from across the CF healthcare system that can solve implementation barriers using ideas that have worked elsewhere. For example, interview data indicated that there were barriers to lack of support across senior management and colleagues (#1 and #2). Participants also reported concerns that adherence support (#2) was only suitable for PWCF without complicated home lives, and this belief hindered healthcare professional’s motivation to discuss adherence. Taking these issues to the collaborative for consideration would allow information about what others think of the behaviour, including how PWCF perceive their care teams using their data (#1) and supporting adherence (#2) to be provided. This would also enable healthcare practitioners to view both behaviours being performed.

### Planning, routine and habits

(#1) Lack of routine and habit was reported by all centres and as such made performing both behaviours effortful and unreliable. Healthcare practitioners lacking established routines or habits for regularly accessing adherence data on CFHealthHub could be identified through website click analytics and self-report. Support would be provided to set achievable targets, focusing on frequency of accessing adherence data. Healthcare professional would be supported to identify prompts or cues in the environment for the behaviour and then action plan performing the behaviour. Prompting rehearsal and repetition in the same context is thought to support habit formation. By supporting healthcare practitioners to build robust habits to behaviour #1, barriers related to ‘forgetting’ and the effort involved in opening CFHealthHub at clinical encounters could be addressed.

(#2) Healthcare practitioners would be supported by CFHealthHub champion to plan when and where they would deliver adherence discussions. It is not expected that ‘habits’ would be created in discussing adherence, as replicability of the behaviour in similar situations is thought to be unlikely.

## Discussion

Median adherence in cystic fibrosis is 30% but without measuring adherence clinical teams cannot determine which patients are deteriorating due to non-adherence and which are deteriorating due to novel pathology. This paper identified the barriers and facilitators for CF healthcare practitioners to implement two behaviours, #1 accessing objective adherence data from the website and #2 discussing adherence with PWCF as part of routine clinical care, through interviews with CF specialist healthcare practitioners. The barriers for each behaviour were mapped to intervention functions and BCTs, using the behaviour change wheel (BCW) [37] which formed the basis of an implementation strategy. As well as identifying the challenges facing CF teams, this paper provides an example of the use of the TDF and BCW to systematically identify facilitators and barriers and derive implementation strategies.

The key goal of successful implementation is to select strategies that are appropriate for the organisations and stakeholders [48, 49] and that can sustain change after the implementation intervention has ended. A key driver of sustainability is likely to be NICE’s adoption of adherence data as a routine quality indicator in CF care [50, 51]. At a team level, interventions such as audit and feedback are thought to produce higher fidelity sustainment [26]. Habit or routine offers a sustainable mechanism for behaviour change in healthcare practitioners [52, 53]. Once established, a routine of accessing adherence data (behaviour #1) might create the habit that would lead to automaticity that would override the requirement for future motivation [52] and may be resilient in the face of increased work related stressors [54]. The implementation package seeks to address fundamental barriers to behaviour #1. If barriers are removed and the behaviour is successfully repeated the establishment of a routine or habit will reduce the burden of behaviour #1 and repetition will be more likely [55]. Future studies should aim to explore whether this strategy supports intervention sustainability.

The same implementation strategy modules can be applied to the complex behaviour of discussing adherence with patients (#2). It is not expected that ‘habit’ would be relevant to behaviour #2, as healthcare practitioners are required to make a judgement about the suitability and content of the discussion on a per person basis. However, planning for the behaviour #2 is important to enable the team to identify who, where and when adherence discussions will be delivered. We hypothesise that reducing the barriers described in this paper could be sufficient to enable increases in adherence discussions within routine practice. Future research should aim to address this question.

While the interviews were conducted on a relatively small number of participants at a limited number of centres, given the narrow study aim, specificity of the sample and the use of established theory [56, 57], the number of interviews is likely adequate to understand common barriers to programme implementation. Multiple coding using a validated framework [34, 58] with the input of an experienced health psychologist increases the trustworthiness of the findings.

Together, the three site function as a ‘scalable unit’ which can be used to assess the necessary requirements for best-practice implementation, and to test the processes and infrastructure needed to achieve full-scale integration of the intervention [59]. A further 19 NHS Trusts expressed an interest in implementing CFHealthHub from June 2019. Conducting this analysis at the outset of the pre-implementation has maximised the opportunity to formulate an implementation strategy that will be applicable during scale up [60]. The next step will be to test this empirically devised implementation strategy and identify the elements which succeed or fail.

## Conclusion

We have devised an implementation strategy to increase and sustain two target behaviours, opening objective adherence data and initiating adherence discussions, both of which are required for implementing the web application ‘CFHealthHub’. The study identified potential pre-implementation facilitators and barriers, reported by CF healthcare practitioners and sensitive to local context. The resulting implementation strategy was developed using the TDF and BCW, demonstrating that the TDF can be used to develop implementation strategies. The success of this specific implementation intervention will be evaluated in future longitudinal research in up to 19 UK CF centres.

## Supplementary Information

**Additional file 1.** CFHealthHub Data Observatory Topic Guide.

## Data Availability

The datasets generated and analysed during the current study are not publicly available due to the potential to identify participants, but are available from the corresponding author on reasonable request.

## Abbreviations

BCT: Behaviour change technique
BCW: Behaviour change wheel
CF: Cystic fibrosis
COM-B: The Capability Opportunity Motivation Behaviour model
MDT: Multidisciplinary team
NHS: National Health Service
PWCF: People with cystic fibrosis
TDF: Theoretical domains framework
UK: United Kingdom

## Footnotes

1 The previous system required multiple steps and did not allow the PWCF or clinician to see the data before the consultation. The device had to be brought to clinic, put into a docking device, the data downloaded and the clinician to manually calculate adherence based on prescription. They would then report back to the PWCF during the clinic appointment.
