# Supplementary material for: Implementing the use of objective medication adherence data in routine clinical practice via the digital CFHealthHub platform: situation analysis and strategy development using the theoretical domains framework
Source: Implement Sci Commun. 2022 Feb 8;3:12. doi: 10.1186/s43058-022-00263-9 (PMC8822811; doi:10.1186/s43058-022-00263-9)
Supplement: Supplementary file 1 — Additional file 1. CFHealthHub Data Observatory Topic Guide. [file 43058_2022_263_MOESM1_ESM.docx]

**
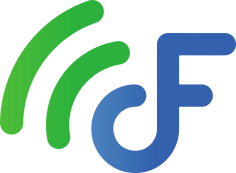
**
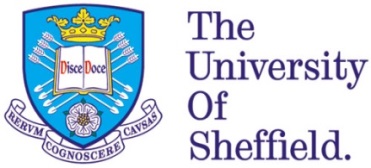


**CFHealthHub Data Observatory Topic Guide**

*Thank you for agreeing to take part in the interview today.*

*Check timing ok*

*Check consent form filled in*

*Are you happy for the interview to be recorded?*

*We’re interested in your views of the CFHealthHub service and how it fits into the care provided in your centre. Please can you confirm your role in the CF team.*

1. What do you know about adherence to nebulisers?
   - [Prompt] What do you think are the most important things for you in your role to know about patient’s adherence?
2. What do you know about your centre's average adherence?
   - [Prompt] Do you think your patients are aware of their adherence?
   - [Prompt] What support do you think is available to patient’s to support adherence behaviours?
3. Do you know what CFHealthHub is and does?
   - [Prompt] Do you know what types of content there are on CFHH?
   - [Prompt] Do you know what tools are available in CFHH?
4. Do you know how to discuss adherence with patients?
   - How confident do you feel about discussing adherence with patients?
   - Have you had any training in techniques to discuss adherence with patients and if so, what?
5. Is discussing adherence something you usually do?
   - [Prompt] When do you discuss adherence and why at that time?
6. How does supporting adherence fit into your role?
   - [Prompt] Who in your MDT do you think CFHH is helpful for?
   - [Prompt] To what extent is the use of CFHH part of your role as a …..?
7. How difficult or easy is it for you to discuss adherence with your patients?
   - [Prompt] Does CFHH make it easier or more difficult to discuss adherence with your patients?
   - [Prompt] What other factors make it easier or more difficult to discuss adherence?
8. How confident are you that using adherence information will increase adherence?
   - [Prompt] Do you think CFHH impact patients adherence? If so how?
   - [Prompt] Do you think it is possible to achieve high adherence in your centre
9. How confident are you that CFHealthHub will improve patient care?
10. Does viewing your patients’ adherence evoke an emotional response?
    - [Prompt] or any other aspects of the project that evoke an emotional response?
11. Do you have systems that you could use for monitoring whether or not you have used CFHH?
    - [Prompt] How do you remember to use CFHH at encounters with patients?
    - [Prompt] How often do you look at other centres metrics?
12. When you look at your data, what would you like to achieve as a CF centre?
13. How difficult or easy is it for you to influence change in your centre?
14. Will using CFHealthHub change the way CF care is delivered in your team?
15. To what extent do physical or resource factors facilitate or hinder you using CFHH? / discussing adherence?
    - [Prompt]How has using CFHH changed your working day / time?
    - [Prompt] Can you tell me more about what resources you’ve used to set up and use CFHH, both individually and as a team?
16. Will using CFHH change your patient’s adherence?
    - [Prompt] What are the other consequences of using CFHH?
    - [Prompt] What are the likely consequences of discussing adherence with your patients?
17. Have you made a decision to use CFHH and to discuss adherence?
    - [Prompt] how often do you want to use it, when, where?
18. To what extent does social influence facilitate or hinder you using CFHH?
    - [Prompt] Do your colleagues discuss adherence and use CFHH?
    - [Prompt] Are there any people in your team who have been particularly influential?
    - [Prompt] Have you got any leadership in your area who is committed to CFHH?
19. Are there incentives to discussing adherence and using CFHH?
    - [Prompt] Have you experienced any positive/negative outcomes as a result of using CFHH?
20. How do you hope CF care develops for patients?

Is there anything else you’d like to say?

**THANK YOU**

*The Data Observatory project is funded by NHS England.*
